# Analysis of the Relationship between Genomic GC Content and Patterns of Base Usage, Codon Usage and Amino Acid Usage in Prokaryotes: Similar GC Content Adopts Similar Compositional Frequencies Regardless of the Phylogenetic Lineages

**DOI:** 10.1371/journal.pone.0107319

**Published:** 2014-09-25

**Authors:** Hui-Qi Zhou, Lu-Wen Ning, Hui-Xiong Zhang, Feng-Biao Guo

**Affiliations:** Center of Bioinformatics and Key Laboratory for NeuroInformation of the Ministry of Education, University of Electronic Science and Technology of China, Chengdu, China; Wayne State University, United States of America

## Abstract

The GC contents of 2670 prokaryotic genomes that belong to diverse phylogenetic lineages were analyzed in this paper. These genomes had GC contents that ranged from 13.5% to 74.9%. We analyzed the distance of base frequencies at the three codon positions, codon frequencies, and amino acid compositions across genomes with respect to the differences in the GC content of these prokaryotic species. We found that although the phylogenetic lineages were remote among some species, a similar genomic GC content forced them to adopt similar base usage patterns at the three codon positions, codon usage patterns, and amino acid usage patterns. Our work demonstrates that in prokaryotic genomes: a) base usage, codon usage, and amino acid usage change with GC content with a linear correlation; b) the distance of each usage has a linear correlation with the GC content difference; and c) GC content is more essential than phylogenetic lineage in determining base usage, codon usage, and amino acid usage. This work is exceptional in that we adopted intuitively graphic methods for all analyses, and we used these analyses to examine as many as 2670 prokaryotes. We hope that this work is helpful for understanding common features in the organization of microbial genomes.

## Introduction

With the wide application of high-throughput sequencing technology, a large number of prokaryotic genomes have been published. This makes it very convenient to mine rules or new patterns from the sequences using comparative analysis methods. Among them, the effect of the genomic GC content on nucleotide or amino acid composition has received special attention [1]–[4]. Especially, some research showed that the GC content in bacterial genomes ranges from about 25% to 75% [5]–[7]. The range is proposed to extend to 0.211 and 0.789 based on theoretical induction [8]. It is a well-known fact that usage of synonymous codons for amino acids is not equal [9]–[11]. Deep analysis [12] showed that codon usage seems compatible with the idea that the genome, not the individual gene, is the unit of selection. That is to say, each gene in a genome tends to conform to its species’ usage of the codon catalog. It was also shown that [13] the genomic GC content of bacteria is related to their phylogeny. The GC content of microorganism genomes is one of the recommended characteristics for the standard description of bacterial species [14], where a low GC difference within 10–12% probably indicates homogeneity and a high GC difference indicates heterogeneity. Indeed, GC content is linked to the codon usage pattern. Previous studies using varied numbers of prokaryotic genomes [15]–[18] showed that the genomic GC content is linearly correlated with the G+C content or single base frequencies of genes. Also, numerous studies illustrated that a similar relationship appeared between the frequency of amino acids and genomic GC content [19]–[22].

Though it was indicated that the GC content has a large impact on base usage at the three positions of a codon, codon usage, and amino acid usage, previous work only considered a limited number of species. Thus, we decided to investigate the influence of genomic GC content on the three usage patterns in a wider range of species. In this paper, the influence is analyzed by regression analysis and intuitively graphic methods within all sequenced bacteria and archaea using the frequencies of bases A, T, C, and G at three codon positions, frequencies of 64 codons, and frequencies of 20 amino acids. We confirmed the existence of a linear relationship between the genomic GC content and amino acid usage [22] using the data of over 2600 sequenced prokaryotic genomes. Also, we confirmed that the genomic GC content has more influence on base usage, codon usage, and amino acid usage than phylogenetic lineage. To do this, we analyzed phylum-divided groups and GC content-divided groups. As expected, the distance variance of the phylum-divided groups is much larger than that of the GC content-divided groups.

## Materials and Methods

### Database

The data used in this paper are sequenced bacterial and archaeal genomes that were available as of September 2013. In total, 2670 prokaryotic genomes along with their annotation information were downloaded from GenBank (ftp://ftp.ncbi.nlm.nih.gov/genbank/genomes/Bacteria). We want to state that theoretically, using a much larger data set may have the possibility to introduce bias in some rare cases although this issue does not appear in this work The corresponding genomic length and GC content information of all these prokaryotic genomes are presented in Table S1.

### Protein coding regions

Protein coding regions were generated using the genome-wide sequence from.fna files, and the information about base location, strand direction, and length were derived from.ptt files. In addition, the coding regions with a length that was not a multiple of 3 were eliminated.

### Base distance, codon distance, and amino acid distance

For notation, we designate matrix 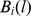 (*l* = 1, 2…12) as base usage vectors with 12 dimensions in the *i*th genome, where B(1) to B(12) indicate A, T, C, and G usage frequencies at the first, second, and third codon positions, respectively. Matrix 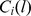 (*l* = 1, 2…64) is codon usage vectors with 64 dimensions in the *i*th genome, and each dimensional vector 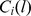 is the frequency of the *i*th codon. Matrix 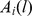 (*l* = 1, 2…20) is amino acid usage vectors with 20 dimensions in the *i*th genome. They are frequencies of the 20 amino acids. 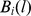, 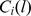, and 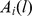 in the *i*th genome is the average of *B*(*l*), *C*(*l*), and *A*(*l*) values among all genes contained. The base, codon, and amino acid usage frequencies are presented in Table S2, S3, and S4, respectively.

These three matrices can be recognized as patterns of base usages, codon usages, and amino acid usages for each species. To compare pattern similarities between different pairs of genomes, the vector distance is required. We used the Euclidean distance as the vector distance in this paper with the following equations (where i corresponds to one genome, and j corresponds to another genome):

Base distance
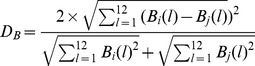



Codon distance
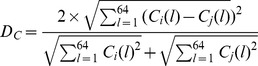



Amino acid distance
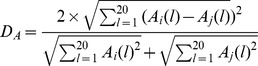



### Linear regression

Linear regression analysis of the relationship model between one or more independent variables and the dependent variable was performed using the least squares function. The function is a linear combination of the model parameters of several regression coefficients, which is named simple linear regression when there is only one independent variable, with the following regression equation:
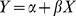



The goodness of fit using analysis of variance includes the following equations:

Sum of squares for error (SSE)
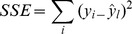



R-square
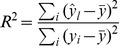



### R software

Related analyses and calculations in our work were accomplished through a tool called R, which is a static and computing language built with language S. R software can be downloaded (http://www.r-project.org/) and used freely.

## Results and Discussion

### Base, codon, and amino acid frequency-based heat maps

**Figure 1:**
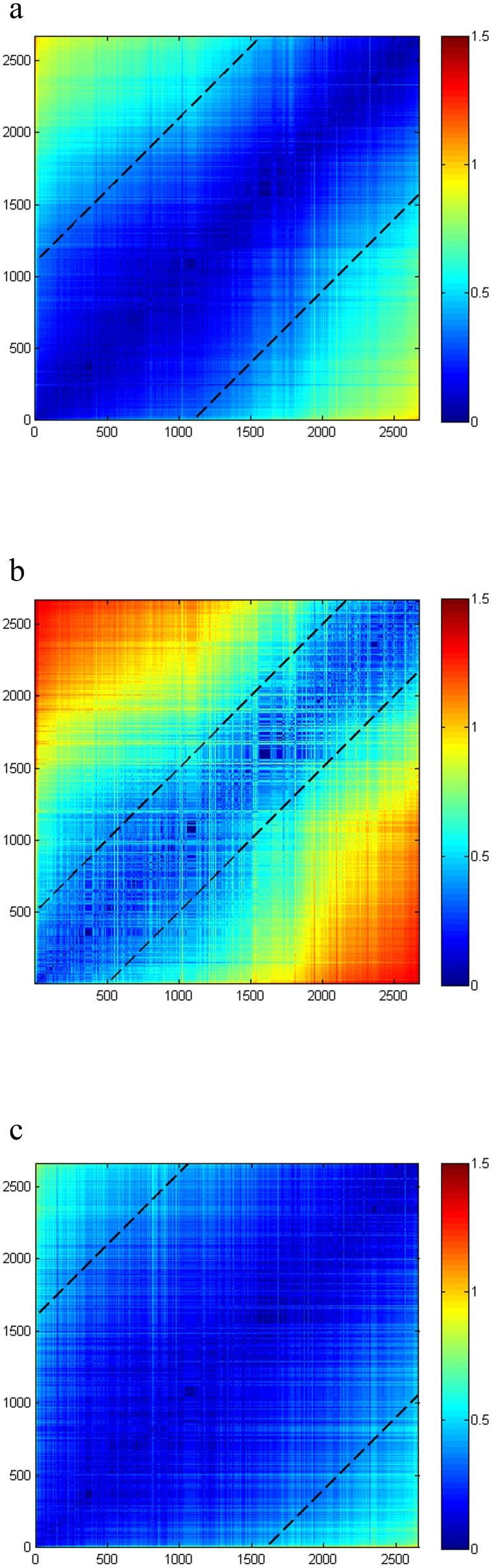
Base, codon, and amino acid frequency-based heat maps. The x-axis and y-axis represent 2670 prokaryotic genomes with GC content arranged from smallest to largest. a) Base frequency-based heat map; maximum base distance = 1.0986. b) Codon frequency-based heat map; maximum codon distance = 1.4199. c) Amino acid frequency-based heat map; maximum amino acid distance = 1.0725.

The base, codon, and amino acid frequency-based heat maps are presented in Fig. 1. The heat maps are based on pair-wise comparisons of the base, codon, and amino acid usage vectors that were created for each two prokaryotic genomes. These vectors, which have been sorted by increasing genomic GC content, were clustered using the vector distance described above. The color button, which is the vector distance, distinguishes the codon, base, and amino acid usage patterns of the two genomes with different GC content. The x-axis and y-axis are not the GC contents of each species; instead, they represent the detailed identity of the species by their GC contents. For example, the genome *Candidatus Zinderia insecticola* CARI, uid52459, whose GC content is 13.5%, is the lowest among the 2670 species and was placed at the leftmost side of the x-axis and the lowest position on the y-axis. The genome *Anaeromyxobacter dehalogenans* 2CP-C, uid58135, whose GC content is 74.9%, is the highest among the 2670 species and was put at the rightmost side of the x-axis and the highest position on the y-axis. On the other hand, each unit length on the x-axis and y-axis represent the same number of genomes.

As seen from the blue areas of the heat maps, within a limited range of GC content difference, the genomes have a small distance between each other. For instance, In Fig. 1a, the first bacterium (*Candidatus Zinderia insecticola* CARI, uid52459) contains a GC content of 0.135388, while genome 441 (*Thermoanaerobacter* X513, uid53065) has a GC content of 0.345191. The GC content difference and base distance between both are 0.209803 and 0.3995, respectively. In Fig. 1b, the 16^th^ genome (*Candidatus Sulcia muelleri* SMDSEM, uid59393) contains a GC content of 0.225952, which has a difference of 0.090564 from the 1^st^ genome. Correspondingly, their codon distance is 0.3683. In Fig. 1c, the 30^th^ genome (*Candidatus Portiera aleyrodidarum* TV, uid195460) has a GC content of 0.246937, which has an amino acid distance of 0.3838 relative to the 1^st^ genome; the GC content between the 1^st^ and the 30^th^ genomes differ by 0.111549. The GC content difference range of the dark blue area (distance smaller than 0.4) of Fig. 1a, b, and c are 0.0000–0.2986, 0.0000–0.1740, and 0.0000–0.4140, respectively.

However, the maximum codon distance is near 1.5 (Fig. 1b) when the genomic GC content gap increases, as shown by the deep red areas. Hence, Fig. 1b indicates that different prokaryotic species with a similar GC content have a similar codon usage pattern. The distance similarity presented in base frequency- and amino acid frequency-based heat maps (Fig. 1a and c). The maximal distances of Fig. 1a, b, and c are 1.0986, 1.4199, and 1.0725, respectively. We found that codon usage has the largest distance with the same GC content difference because the dark blue area (distance smaller than 0.4) is the least among the three figures, and base usage has the least distance as indicated by the largest blue area. The differences found among the three heat maps is possibly attributed to increasing vector dimensions among base usage, amino acid usage, and codon usage and the fact that all amino acids except Met and Trp are encoded by more than one codon. The undisputed indication is that base, codon, and amino acid usage change with genomic GC content.

### Base distance, codon distance, and amino acid distance vs. GC content difference

**Figure 2:**
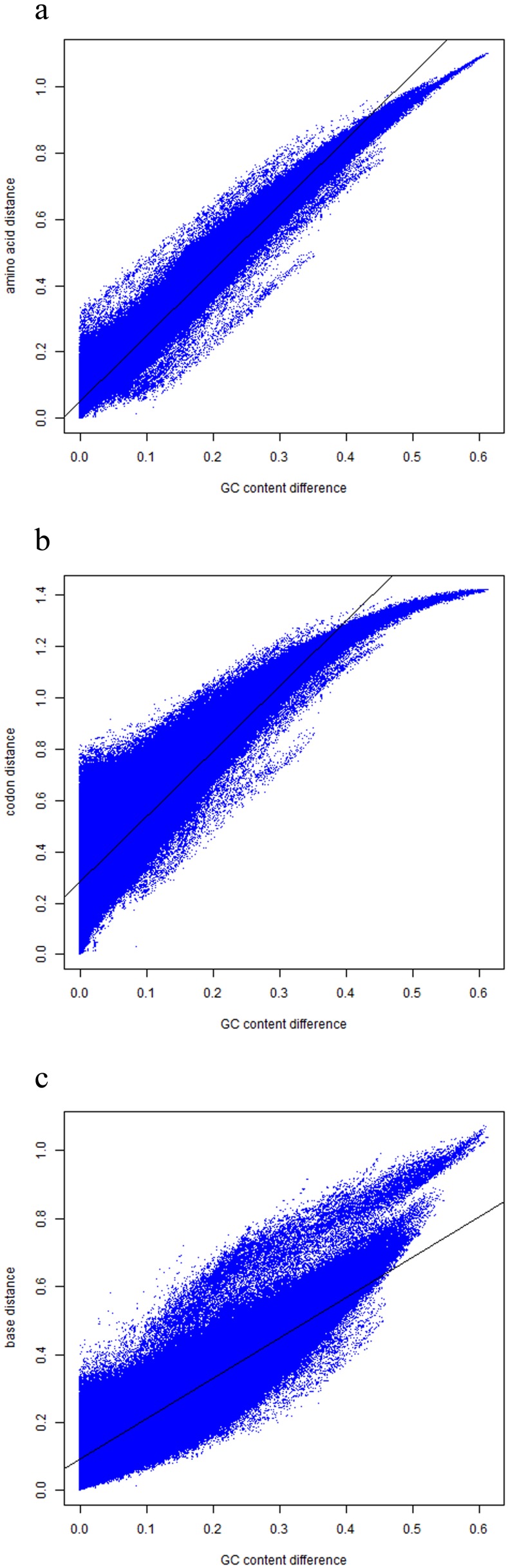
Vector distance of two genomes plotted against their GC content difference. a) Y: Base distance; X: D-value of GC content. b) Y: Codon distance; X: D-value of GC content. c) Y: Amino acid distance; X: D-value of GC content.

To analyze how the base usage, codon usage, and amino acid usage change with genomic GC content, the frequency vector distances were plotted against the genomic GC content difference in Fig. 2. The linear regression model for each scatter plot was established, and the corresponding results are listed in Table 1.

**Table 1:** Results of least squares fitting between vector distance of genome pairs and their GC content difference.

	Slope	Intercept	SSE	R-square
**Base distance**	1.968	0.05285	4332	0.9732
**Codon distance**	2.532	0.2871	2.556e+004	0.9108
**Amino acid distance**	1.185	0.09203	1.356e+004	0.8069

The linear regressions of all the three models are obvious and positive (the slopes are 1.968, 2.532, and 1.185), and the R-square of the base distance regression is the highest (0.9732). This means that the genomic GC content has a stronger impact on base usage than the other two usages, which have R-square values of 0.9108 and 0.8069. However, the points are more dispersive in the amino acid distance regression than others. This phenomenon indicates that the contribution of the genomic GC content to the amino acid composition is the weakest. Such a situation may be attributed to the fact that amino acid bias is associated with base bias [2], [23] and is not a direct effect of the GC content.

In Fig. 2, we noticed that the ranges of the base usage, codon usage, and amino acid usage distance corresponding to a specific range of GC content differences decrease monotonically with an increasing GC content difference. For instance, in Fig. 2a, among the whole points, the GC content difference between 0.0000 and 0.0100 has a range of base usage distance from 0.0000 to 0.1802, which contains 172,973 points. However, a GC content difference between 0.6000 and 0.6100 has a range only from about 1.0815 to 1.0915, which contains only 35 points (Fig. 2a). In other words, pairs with a small GC difference may have a relatively large composition distance, but pairs with a large GC difference do not have a small composition distance. We believe that the large distance between some pairs with a small GC difference is caused by some composition differences among species that are not only determined by GC content. For example, previous results indicated that genes with a high GC content have a high gene expression level [24], [25]. Furthermore, codon usage can maintain a force balance between mutational bias and translational selection [26]. Thus, gene expression level, gene function, and origination are additional fundamental factors that shape the pattern of biased codon usage.

### Base, codon, and amino acid frequencies vs. genomic GC content

**Figure 3:**
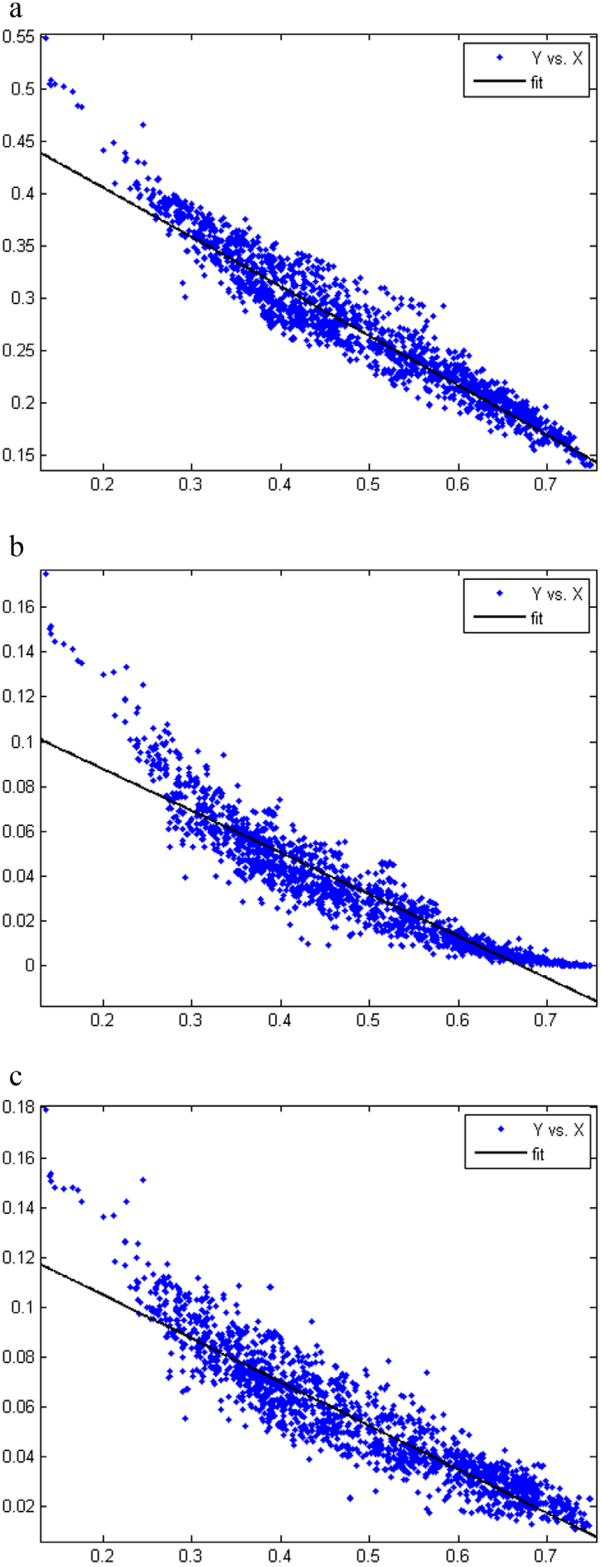
Base, codon, and amino acid frequencies of 2670 prokaryotic genomes plotted against the genomic GC content. a) Y: Base A frequency at the first codon position; X: genomic GC content. b) Y: Codon AAA frequency; X: genomic GC content. c) Y: Amino acid Lys frequency; X: genomic GC content.

To directly analyze whether the base, codon, and amino acid usage are correlated with the genomic GC content, we checked the usages of all bases (4 types×3 positions of a codon = 12), codons (64 types), and amino acids (20 types). Here, we picked out the frequencies of base A at the first codon position, codon AAA, and amino acid Lys, which is translated from AAA, as an example. The frequencies against genomic GC content are shown in Fig. 3, and the results of fitting are shown in Table 2. As seen from Fig. 3 and Table 2, we found that 1) the usage of A at the first codon position, AAA, and Lys decreased almost linearly with increasing genomic GC content, and 2) genomes with both a high GC content and a low GC content adopt a similar pattern. Furthermore, our work shows that the almost linear relationship between the genomic GC content and the base usage, codon usage, and amino acid usage is consistent across all sequenced genomes of different species.

**Table 2:** Results of least squares fitting between base, codon, and amino acid frequencies and the genomic GC content.

	Slope	Intercept	SSE	R-square
**A_1_** *	–0.4728	0.5011	0.9880	0.9091
**AAA**	–0.1865	0.1251	0.2231	0.8733
**Lys**	–0.1748	0.1399	0.2287	0.8549

*A_1_ denotes base A at the first codon position.

To obtain a quantitative measurement of the frequencies of bases, codons, and amino acids, we also calculated the slope of the best-fitting line for each scatter plot. The slope for base A is −0.4728, which means that if one bacterial genome has a 10% higher GC content than another, the percentage of base A at the first codon position would decrease approximately 4.728%. The results of codon AAA frequencies and amino acid Lys frequencies showed a similar, but smaller, effect with slopes of −0.1865 and −0.1748, respectively. Lightfield et al. reported that the usage percentage of amino acids encoded by three low-GC codon families including Lys and genomic GC content of the representative genomes showed a negative linear relationship, which was roughly consistent with our work [22].

### Phylum and GC content

Although the results described above indicate that the GC content has a strong biased mutation pressure, we need to know whether the pressure is stronger than the phylogenetic distribution.

**Figure 4:**
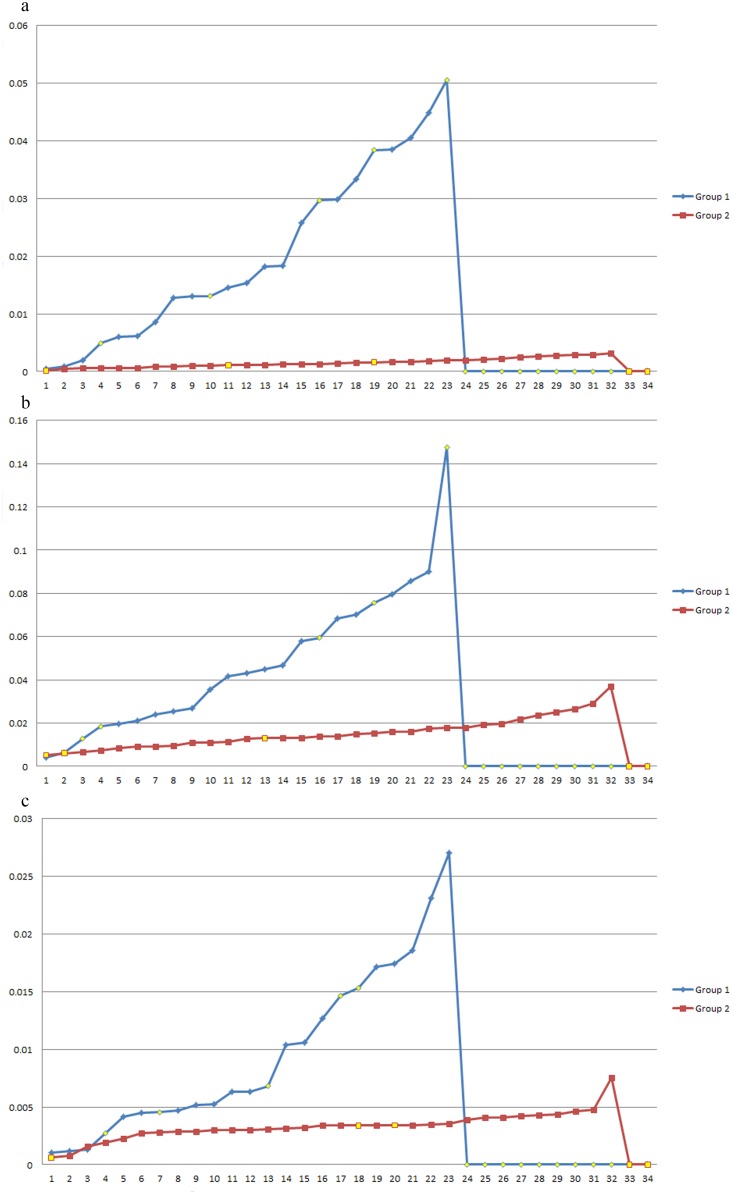
Line charts of variances of base distance, codon distance, and amino acid distance for phylum-divided groups and genomic GC content-divided groups. Group 1: the data were divided based on phylum; Group 2: the data were divided based on genomic GC content. a) Y: Base distance variances; X: sections. b) Y: Codon distance variances; X: sections. c) Y: Amino acid distance variances; X: sections.

In the next analysis, all 2670 bacterial and archaeal genomic sequences were divided into 34 sections based on phyla (Group 1) and genomic GC content (Group 2). In Group 1, each section includes one phylum, and five unclassified bacteria and archaea (*Halophilic archaeon* DL31, uid72619; *Candidatus Cloacamonas acidaminovorans* Evry, uid62959; *Candidatus Saccharobacterium alaburgensis*, uid203361; *Candidatus Methylomirabilis oxyfera*, uid161981; and *Thermobaculum terrenum* ATCC BAA-798, uid42011) were excluded. In Group 2, the GC content range of each section was averaged, which has a range among x = (0.749053−0.135388)/34 (genomic GC content ranges from 0.135388 to 0.749053). Thus, the GC content of the *N*th section has a range from 0.135388 to 0.135388+*N*×x. Number of genomes in each section of the two groups are shown in Table S5 and S6, respectively. The base distance, codon distance, and amino acid distance were investigated for every section except the sections with data from only one and two genomes. Thus, if there are data for M (M>2) genomes in one section, then there are M×(M-1)/2 distance data. The variance of the M×(M-1)/2 distance data was calculated for each section to reflect the impact of the phylum or GC content on the base, codon, and amino acid usage. When M≤2, the variance value of this section was set to 0. The line charts of variances of the two groups were plotted, and nonzero values were plotted increasingly in Fig. 4.

Fig. 4 shows that the base distance variances of Group 1 vary widely from 0.000477 to 0.050552. Compared with Group 1, the base distance variances of Group 2 range within a smaller scale from 0.000169 to 0.003120. Additionally, the codon distance variances in Group 1 and Group 2 range from 0.004812 to 0.147581 and from 0.005235 to 0.036949, and the amino acid distance variances of the two groups are from 0.001018 to 0.027048 and from 0.000627 to 0.007536, respectively. Nevertheless, several particularly high values appear in both Group 1 and Group 2. This may be caused by the fact that the genome sequence data were too few in these sections. We marked data with fewer than 5 sequences as yellow points in Fig. 4 so that these data did not affect the analysis.

A previous report indicated the relationship between the bacterial genomic GC content and phylogeny through a phylogenetic tree [13]. Here, our results from graphic analysis illustrate that GC content is more important than phylogenetic lineages in general for their base, codon, and amino acid usage biases because the variances from the GC content are much smaller than those from phylogenetic lineages. This conclusion is consistent with that obtained for codon usage patterns in microbial genomes with a high GC content [17] and is also consistent with that obtained for amino acid frequencies [22]. However, the conclusion in this paper is based on a much larger data set and different analyzing methods.

## Conclusions

In this paper, we analyzed the base usages, codon usages, and amino acid usages with respect to the genomic GC contents of a large number of prokaryotic genomes. Our work has demonstrated that in prokaryotic genomes: a) base usage, codon usage, and amino acid usage changes with GC content with a linear correlation; b) the distance of each usage has a linear correlation with the GC content difference; and c) the GC content has a larger impact on base usage, codon usage, and amino acid usage than the phylogenetic lineage. We believe that our work will be helpful to better understand the role that GC content plays in prokaryotic genomes.

## Supporting Information

Table S1 **Genomic length and GC content of all samples included in the analyses.**
(XLS)

Table S2 **Mean usage frequencies of base A, T, C, and G at the first, second, and third codon positions of all samples included in the analyses.**
(XLS)

Table S3 **Mean usage frequencies of 64 codons of all samples included in the analyses.**
(XLS)

Table S4 **Usage frequencies of 20 amino acids of all samples included in the analyses.**
(XLS)

Table S5 **Number of genomes, mean and variance of the distance for nucleotide frequencies, codon usages and amino acid compositions in each section of Group 1, that is to say, each section corresponds to one phyla.**
(XLS)

Table S6 **Number of genomes, mean and variance of the distance for nucleotide frequencies, codon usages and amino acid compositions in each section of Group 2, that is to say, each section corresponds to one specific G+C interval.**
(XLS)
